# Uncovering the differentiated impacts of carbon neutrality and clean air policies in multi-provinces of China

**DOI:** 10.1016/j.isci.2024.109966

**Published:** 2024-05-11

**Authors:** Meng Xu, Minghao Wang, Mengdan Zhao, Zhixiong Weng, Fan Tong, Yujie Pan, Xin Liu, Yang Xie

**Affiliations:** 1 School of Management, Wuhan Institute of Technology, Wuhan 430205, China; 2 China Institute of Marine Technology and Economy, Beijing 100081, China; 3 School of Economics and Management, Beihang University, Beijing 100191, China; 4 Institute of Circular Economy, Beijing University of Technology, Beijing 100124, China; 5 Laboratory for Low-carbon Intelligent Governance, Beihang University, Beijing 100191, China; 6 Peking University Ordos Research Institute of Energy, Ordos City, Inner Mongolia 017000, China; 7 College of Environmental Sciences and Engineering, Peking University, Beijing 100871, China; 8 Energy Foundation China, Beijing 100004, China

**Keywords:** Economics, Energy policy, Energy sustainability, Public health

## Abstract

Ambitious action plans have been launched to address climate change and air pollution. Through coupling the IMED|CGE, GAINS, and IMED|HEL models, this study investigate the impacts of implementing carbon neutrality and clean air policies on the energy-environment-health-economy chain in the Beijing-Tianjin-Hebei-Henan-Shandong-Shanxi region of China. Results show that Shandong holds the largest reduction in energy consumption and carbon emissions toward the 1.5°C target. Shandong, Henan, and Hebei are of particularly prominent pollutant reduction potential. Synergistic effects of carbon reduction on decreasing PM_2.5_ concentration will increase in the future, specifically in energy-intensive regions. Co-deployment of carbon reduction and end-of-pipe technologies are beneficial to decrease PM_2.5_-related mortalities and economic loss by 4.7–12.9% in 2050. Provincial carbon reduction cost will be higher than monetary health benefits after 2030, indicating that more zero-carbon technologies should be developed. Our findings provide scientific enlightenment on policymaking toward achieving carbon reduction and pollution mitigation from multiple perspectives.

## Highlights

• Comprehensive effects of climate change mitigation and pollution control are assessed
• Joint decreasing degrees in SO_2_, NO_X_, and PM_2.5_ are significantly higher than that in VOC
• Synergistic effects of carbon reduction on decreasing PM_2.5_ concentration will increase
• More zero-carbon technologies should be developed toward reducing the costs

## Graphical abstract

**Figure:**
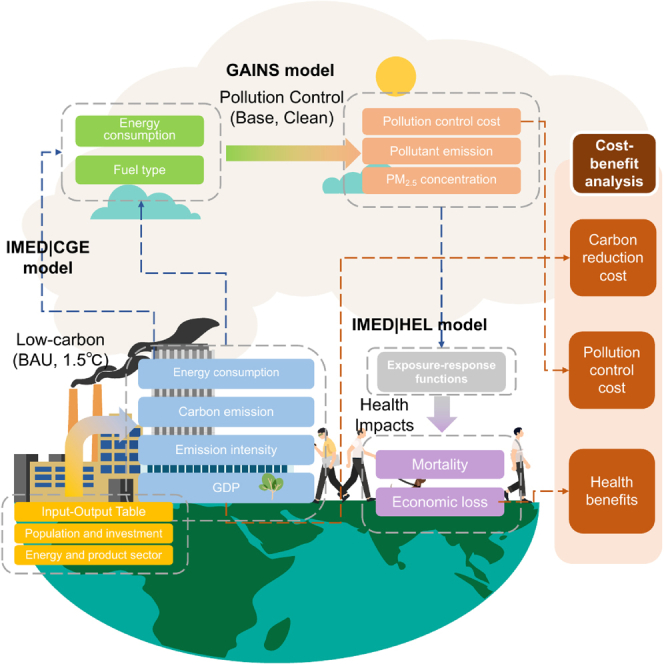

## Introduction

Extensive use of fossil fuels not only promotes fast economic development, but also causes significant carbon emissions, leading to climate change and greenhouse effects.^1^^,^^2^ It has been reported that global CO_2_ emissions increased by 150% during 2000–2019, and CO_2_ concentration will rapidly double by the 22nd century if emission levels remain unchanged.^3^ To address this problem, the Paris Agreement, issued by the Parties to the United Nations Framework Convention on Climate Change, is launched, aiming to “hold the increase in global average temperature well below 2°C above pre-industrial levels and pursue efforts to hold the increase to 1.5°C above pre-industrial levels”.^4^ With the acceleration of industrialization, China has also become the world’s largest CO_2_ emitter and energy consumer.^5^^,^^6^^,^^7^ It is also one of the countries with poor environmental performance in terms of ecological footprint and load capacity factor.^8^ It is reported that China’s ecological footprint exceeded its biocapacity by 302% in 2017.^9^ Tremendous CO_2_ emissions have led to severe environmental problems,^10^ therefore it announced to realize the carbon neutral goal by 2060, which will meanwhile promote the high-quality development of the social economy. However, the local CO_2_ emissions are still on the rise. Therefore, great efforts need to be redoubled to achieve deep carbon reductions.

Apart from the great pressure to decrease carbon emissions, China also faces severe air pollution. The estimated average PM_2.5_ concentration reached 29.0 μg/m^3^ in 2022, which was approximately six times the World Health Organization standard of 5 μg/m^3^. The severe air pollution problems China faces pose significant threats to public health. It is estimated that air pollution-related morbidity and mortality accounts for more than 20% of the total nationwide, and in some serious locations such as Beijing and its surrounding areas, reaching around 40%.^11^ Many studies have further concluded that severe PM_2.5_ pollution cause premature death and morbidity from chronic or acute diseases, such as respiratory and cardiovascular illness, which meanwhile resulting in economic burden, such as increased medical expenditure and reduced labor productivity.^12^^,^^13^ In order to abate air pollution and combat related adverse effects, a series of end-of-pipe management have been carried out,^14^ such as the Air Pollution Prevention and Control Action Plan and the Three-Year Action Plan for Winning the Blue Sky War. Moreover, the goal of these atmospheric governance have been increasingly stringent, from limiting single pollutant emission to systematically controlling multiple pollutants, and then to improving air quality.^15^ Assessing how these carbon reduction and clean air policies will affect the energy-environment-human-economy chain are critical to formulating green low-carbon development paths.

Studies have assessed the impacts of achieving different climate targets at the national level^16^^,^^17^ or a single region level,^18^ while few at the spatially differentiated perspective among multi-regions. Moreover, the combined effects under the dual goals of climate change mitigation and atmospheric pollution abatement are poorly considered. For example, Zhang et al.^19^ present that, to achieve the carbon neutrality target on schedule, national proportion of non-fossil fuels in primary consumption and electricity in terminal consumption should be increased to exceed 80% and 70%, respectively, in 2060. Luo et al.^3^ explore the CO_2_ reduction potential from deep decarbonizing the energy system in Sichuan, an area with rapid economic development in China. Huang et al.^20^ reveal the synergetic outcomes of atmospheric pollution abatement actions on decreasing carbon emissions in China. These studies have provided essential insights into the impacts of achieving different green low-carbon development targets. It should be noted that most of them haven’t fully taken into account the characteristics of individual regions, including the local industrialization process and socio-economic development level.^21^^,^^22^ Therefore, locally tailored researches need to be carried out to aid policymakers develop feasible green low-carbon strategies.

In addition, sectoral analysis for the effects toward achieving climate goals is mainly concentrated on a certain individual one,^23^^,^^24^ while few make detailed comparisons among various sectors. For example, Wu et al.^25^ simulate the residential CO_2_ emissions under different shared socioeconomic pathways (SSP). Ren et al.^26^ explore appropriate transformation paths for China’s iron and steel industry toward the carbon neutral goal. Zhang and Hanaoka^27^ examine the carbon reduction potential in the transportation sector to achieve carbon neutrality. It can be found that atmospheric emissions of the whole energy system, especially CO_2_, have not drawn enough attention, resulting in difficulties in accurately capturing emission changes brought by low-carbon transformation. Therefore, there is an urgent need for a more comprehensive assessment of different energy-related sectors. Against these backgrounds, as far as we know, no previous study has been conducted to compare the multifaceted impacts of achieving both the climate and clean air targets on areas with spatial disparities in a detailed sectoral and temporal characterization.

**Figure 1:**
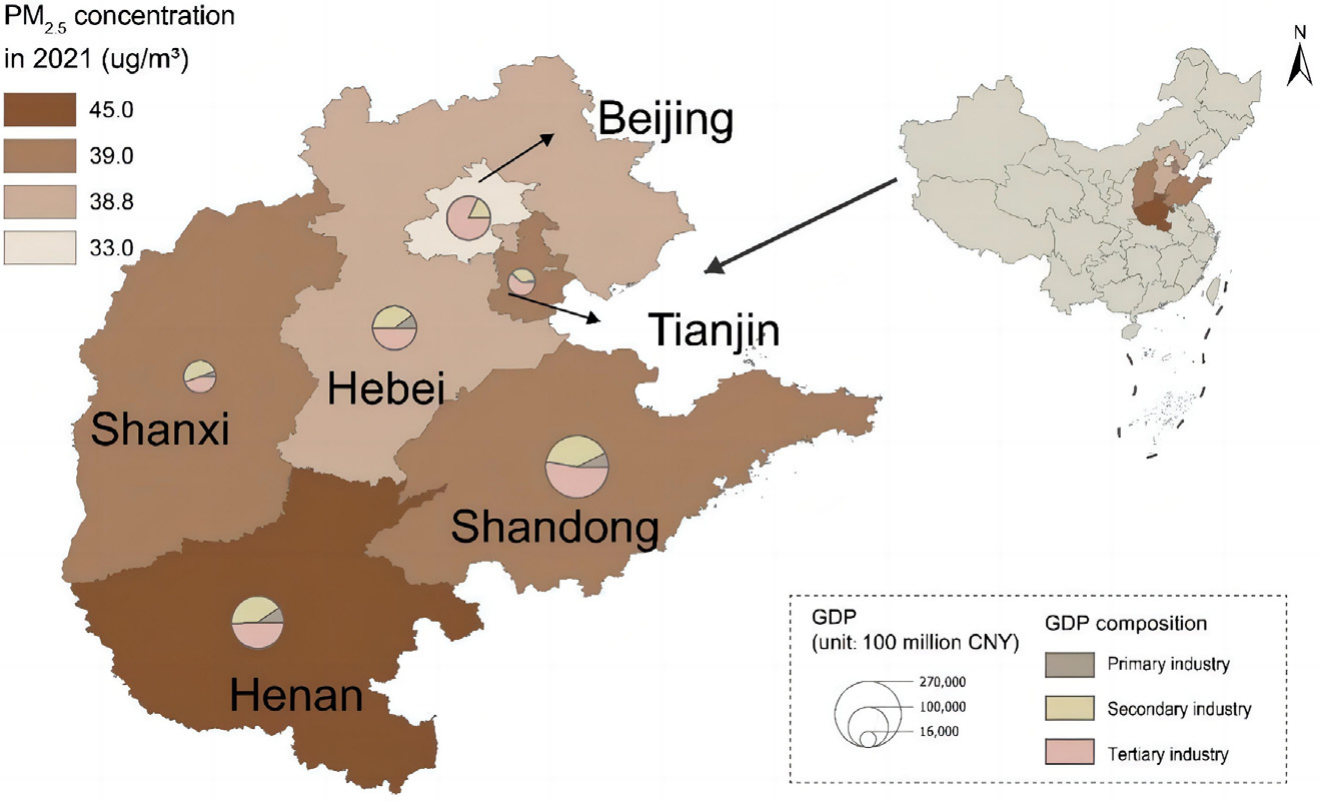
Targeted provinces/municipalities in this study

In this study, we adopt the Beijing-Tianjin-Hebei-Henan-Shandong-Shanxi region (Figure 1) as the research object for several reasons. Firstly, this region, although covering only 7.2% of China’s land area, consumes 33% of national coal, thus facing great burden of energy decarbonization and atmospheric emission reduction.^28^^,^^29^ Secondly, it is in line with the national policy orientation. As put forward in the “Beijing-Tianjin-Hebei and Surrounding Areas Air Pollution Prevention and Control Work Plan in 2017”, the significant scope of “2 + 26” consists of 2 municipalities (Beijing and Tianjin) and 26 cities in Hebei, Shanxi, Shandong, and Henan. Thirdly, this region has made tremendous effort toward green low-carbon transition in the power, industry and transport sectors,^30^ while the integrated impacts of implementing these actions remain unexplored. In this regard, this research concentrates on the Beijing-Tianjin-Hebei-Henan-Shandong-Shanxi region.

Therefore, to fill the previous scientific knowledge gaps, we construct an approach by coupling the IMED|CGE (integrated model of energy, environment and economy for sustainable development|computable general equilibrium), GAINS (greenhouse gas and air pollution interactions and synergies) and IMED|HEL (health) model to systematically and progressively simulate the energy-environment-health-economy impacts under different climate and end-of-pipe management scenarios among the six provinces by 2050. Specifically, the results provide insights on five fronts: the changes to final energy demand and energy consumption structure compliant with climate targets, the energy-related carbon emission reductions and contributions of different influencing sectors, the pollutant emission reduction potential under climate change mitigation and clean air actions, the improvement in air quality and the degree they benefit human health, and the corresponding cost-benefits. The main novelties and contributions are listed as follows: (1) constructing a reliable and accurate model system for provinces with heterogeneity in economic level, energy structure, and natural resource endowment. It improves the accuracy and precision of scenario simulation through parameter localization and optimization and the policy recommendations put forward are more practical and applicable compared with existing researches. (2) Systematically evaluate the comprehensive performance of the whole chains of energy-environment-health-economy under different scenarios, which is of vital importance for promoting health-driven collaborative governance of air pollution and climate change. (3) Carbon emissions of the whole energy system have been assessed, which makes it possible to capture the exact reductions caused by technological transformation in a detailed sectoral, spatial, and temporal characterization.

Rest of the study is arranged as follows. Section 2 illustrates the methodology and establishes different scenarios. Section 3 displays the evaluation outcomes from the energy, environment, health, and economy aspects accordingly. Section 4 reports the discussion and comparison with other studies. Section 5 summarizes the conclusions.

## Results

### Energy consumption

**Figure 2:**
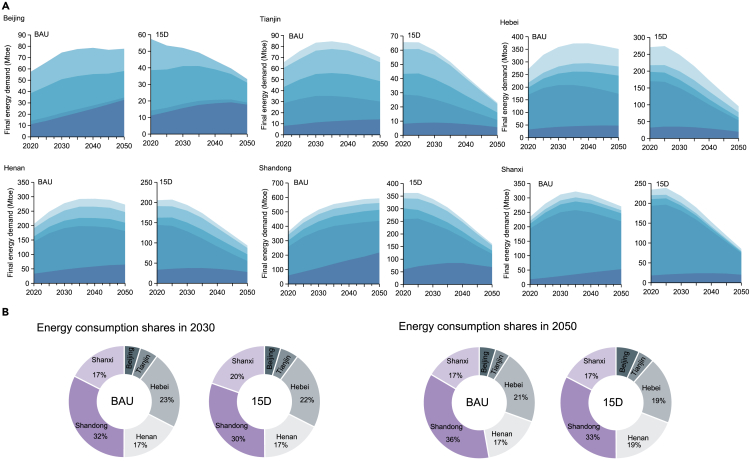
Provincial energy consumption under different climate scenarios (A) Final energy demand transformations under different scenarios from 2020 to 2050; (B) share of total energy consumption for each region under different scenarios in 2030 and 2050.

Figure 2A and Table S1 show total energy consumption by province during 2020–2050. Specifically, the province with the highest total energy consumption in 2020 was Shandong (362.6 Mtoe), followed by Hebei (271.1Mtoe), Shanxi (234.3 Mtoe), and Henan (205.6 Mtoe). Energy consumption in Beijing and Tianjin presented to be at lower levels comparing to these areas, amounting to 57.4 and 65.5 Mtoe, respectively, in 2020. Under the BAU (Business-As-Usual) circumstance, energy consumption in Shandong shows a continually increasing trend from 2020 to 2050, reaching 593.5 Mtoe in 2050. In contrast, other provinces have energy consumption peaking at 78.6 Mtoe (Beijing) to 373.5 Mtoe (Hebei) during 2035–2040, and then slowly declining. From the perspective of growth rates, Shandong grows the fastest, increasing by 163.7% in peak value compared with 2020, followed by Henan (142.5%), Hebei (137.8%), Shanxi (137.5%), Beijing (136.8%) and Tianjin (129.0%). The high growth rate in Shandong is closely associated with the fast development of energy-intensive industries. Besides, between 2020 and 2050, consumption inequalities among different regions gradually increase, manifested by the widening consumption gaps. For example, in 2030, energy consumption in Shandong will be 6.9 and 1.4 times higher than Beijing and Hebei, respectively, while the multiples will increase to 7.6 and 1.7 in 2050 (Figure 2B). In terms of coal consumption, Shandong is the region with largest consuming amount, which is as a result of the heavy-industry dominated economic structure.^31^ Shanxi is rich in coal resources, so coal is more commonly used as an energy choice. Specifically, in BAU, coal consumption will reach 165.8 Mtoe in 2050, 15.5 times than of oil and 6.0 times than of gas. Hebei and Henan are the top coal consumers, consuming 126.0 and 116.1 Mtoe, respectively, in 2050. Beijing holds significant growths in electricity consumption, with the proportion increasing from 19.4% in 2020 to 42.1% in 2050.

In the 15D scenario, all the six provinces will experience an energy consumption with the peaking value 22.6–38.9% lower compared with BAU and the peaking time occurring during 2020–2025 (Figure 2A). It can be seen that Shandong remains the largest energy consumption province, estimating to reach 160.8 Mtoe in 2050, followed by Hebei (95.5 Mtoe), Henan (93.7 Mtoe), Shanxi (85.1 Mtoe). They are also the top four provinces in terms of the reduction percentage in total energy consumption, with cumulative decreases of 65.2%, 64.3%, 55.6%, and 54.8% from 2025 to 2050. Beijing and Tianjin have relatively low energy consumption levels, with values of 33.1 and 23.0 Mtoe in 2050, respectively. In terms of coal consumption, compared with BAU, the province with the highest coal reduction in the 15D scenario is Shandong, accounting for 167.1 Mtoe (35.5%) of total consuming reductions in 2050. Corresponding, other top three provinces with large reductions are Shanxi (111.8 Mtoe in 2050), Hebei (92.0 Mtoe in 2050), and Henan (88.7 Mtoe in 2050). In terms of oil, Beijing and Shanxi have the highest oil consumption reduction ratios, and it will decrease by 53.4% and 45.8% in 2030 and 89.3% and 79.1% in 2050, respectively, under the 15D scenario compared with BAU. In terms of gas, Shandong has the highest embodied consumption during the whole evaluating period. Meanwhile, its gas consumption reduction rate increase from 48.1% in 2030 to 82.9% in 2050 in 15D scenario compared with BAU. In 2050, Hebei surpasses Shandong, becoming the region with the highest reduction rate by 87.9% in gas consumption. Toward achieving climate goals, fossil fuels will be gradually phased out, meanwhile electricity consumption increases.^32^ Specifically, the provincial levels of terminal electrification in 2020 ranged from 7.8% (Shanxi) to 19.4% (Beijing) and the 1.5°C target will facilitate this process. The region with the highest proportion of electricity consumption is Beijing, accounting for 30.2% in 2030 and 53.7% in 2050, respectively (Figure 2A). Other top three areas with high electrification rates are Shandong (43.1% in 2050), Henan (30.0% in 2050), and Tianjin (25.0% in 2050). In addition, top regions holding the largest growth rates in electricity consumption are Beijing, Hebei, and Shandong, with increases by 11.6%, 7.1%, and 6.2% in 2050 in 15D scenario compared with BAU, respectively. In the future, to meet stringent climate targets, more alternative fuels need to be applied and the shares of electric energy equipment ought to be increased.

### Carbon emissions

**Figure 3:**
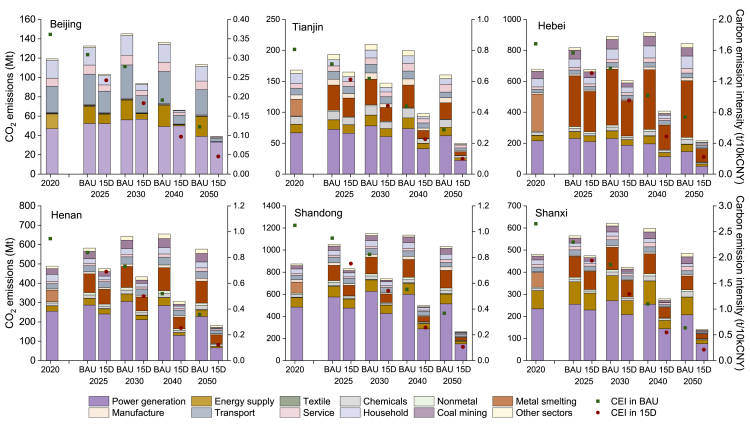
Provincial carbon emissions during 2020–2050 under different climate scenarios (Bars refer to the left Y axis and scatters refer to the right Y axis.)

Figure 3 and Table S2 present the energy-related carbon emissions under different scenarios during 2020–2050. Regional distributions reveal that the top two CO_2_ emitters are Shandong (e.g., 874.0 Mt in 2020) and Hebei (678.0 Mt in 2020) due to high levels of industrialization, while Beijing (119.2 Mt in 2020) and Tianjin (168.2 Mt in 2020) have relatively low CO_2_ emissions. The results are comparable to the emissions estimated by MEIC (MEIC, 2021). Under the BAU scenario, all the six provinces could hardly peak their carbon emissions before 2030. Specifically, the earliest peaking might be observed for Beijing and Tianjin, with estimates of 145.1 and 209.5 Mt in 2030, respectively. The remaining four provinces (Hebei, Henan, Shandong, and Shanxi) would likely to peak in 2035, where the largest peaking emissions (1174.2 Mt) are found in Shandong. From 2020 to their respective peak years, Henan’s emissions will increase the most, reaching 36.7%, while Beijing’s emissions will increase the least, at 21.7%. Then, the emissions will drop by 8.4% (Hebei) −23.4% (Shanxi) toward 2050. Unlike the BAU scenario, the 15D scenario allows the six regions to peak emissions at lower levels before 2025. Specifically, Shandong, Hebei, Henan, Shanxi, Tianjin, and Beijing are expected to peak around 2020 with emissions of 874.0 (decreased by 25.6% compared with the peaking value in BAU), 678.0 (26.4%), 488.6 (26.8%), 480.6 (23.9%), 168.2 (19.7%), and 119.2 (17.8%) Mt, respectively. Additionally, in 2050, their respective carbon emissions under the 15D scenario will be reduced by 769.3 (comparable to a 74.6% reduction in BAU), 628.8 (74.5%), 394.7 (68.5%), 344.5 (71.0%), 110.7 (68.9%), and 74.4 (65.7%) Mt, respectively. In summary, there are obvious regional differences in the peaking level and time of carbon emissions for meeting climate goals, leading to different contributions to carbon reductions.

From the perspective of carbon emission composition, except for Hebei, sector emitting the most is the power generation (shown by the bars in Figure 3). In 2020, it accounted for 31.7% (Hebei) −55.3% (Shandong) of the total emissions. It can be found that although the absolute levels of carbon emissions from power generation continuously decrease in the 15D scenario, it remains a major CO_2_ emitting sector in most regions by 2050. Furthermore, at regional level, to achieve the 1.5°C goal, the province with the highest carbon reductions in power generation is Shandong, reaching 199.9 Mt (accounting for 49.2% of the total carbon reductions in 15D scenario compared with BAU) in 2030, followed by Henan (94.1 Mt, 44.8%) and Shanxi (62.8 Mt, 31.5%). In 2050, reductions by 362.6 Mt (45.6%), 162.1 Mt (41.1%), and 130.3 Mt (37.8%) will be achieved accordingly. As the largest metal smelting base in China, Hebei experiences great carbon emissions in the metal smelting sector. Specifically, it is predicted that the sectoral CO_2_ emissions will reach 382.2 and 366.0 Mt by 2030 and 2050 in BAU, respectively, consisting of 41.4% and 43.4% of the total carbon emissions in Hebei. Besides, carbon reductions by 279.7 Mt in this sector of Hebei will be obtained in 2050 toward the 1.5°C target, followed by Shandong (133.8 Mt) and Henan (67.6 Mt). These reductions accordingly account for 44.5%, 17.4%, and 17.1% of the total provincial carbon reductions in 2050. CO_2_ reductions from road transport sector will be most apparent in Beijing, reaching 23.0 Mt (31.0%) in 2050, followed by Shandong (19.8 Mt, 2.6%) and Henan (18.2 Mt, 4.6%). To sum up, there are obvious heterogeneity in emission levels and reduction contributions of different sectors among the six regions, which will be both a challenge and an opportunity. By taking more targeted strategies, considerable environment benefits will be obtained in the future.

In addition, as shown by the scatters in Figure 3, the carbon emission intensity (CEI) in either scenario shows a downward trend and the 15D scenario experiences a sharper decline. For example, CEI of Shandong will decrease from 1.04 tCO_2_/10 thousand RMB (Renminbi) in 2020 to 0.37 tCO_2_/10 thousand RMB in BAU and 0.11 tCO_2_/10 thousand RMB in 15D by 2050, with decreases of approximately 65.0% and 89.9%, respectively. At regional level, significant spatial differences exist between the features of carbon emissions and CEI. Beijing and Tianjin experience low levels of carbon emission, as well as low CEI (0.18 and 0.44 tCO_2_/10 thousand RMB in 2030 in 15D scenario, respectively). Shanxi and Hebei are characterized with relatively lower carbon emissions compared with Shandong but higher CEI (1.29 and 0.95 tCO_2_/10 thousand RMB in 2030 in 15D scenario, respectively), indicating small and lag-behind economy. On the contrary, Shandong features the highest CO_2_ emissions while relatively lower CEI (0.54 tCO_2_/10 thousand RMB in 2030 in 15D scenario) compared with Shanxi and Hebei. Additionally, from 2020 to 2050, the reduction of CEI in Shanxi and Hebei, amounting to 2.4 and 1.5 tCO_2_/10 thousand RMB, are significantly higher than that of Beijing (0.32 tCO_2_/10 thousand RMB) and Tianjin (0.71 tCO_2_/10 thousand RMB). This may be due to that Shanxi and Hebei sharply cut the proportion of carbon-intensive production toward realizing climate goals. It is consistent with Wu et al.*,*^25^ which concludes that Hebei could effectively reduce CEI in short term through increasing capital and labor input to replace energy consumption.

### Atmospheric pollutant emissions

**Figure 4:**
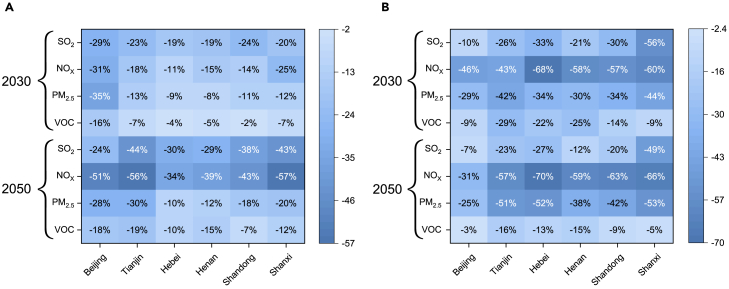
Pollutant emission reduction effect (A) between the BAU_Base and 15D_Base scenario and (B) between the 15D_Base and 15D_Clean scenario

The implementation of CO_2_ reduction and clean air strategies will decrease pollutant emissions at different degrees.^20^ As shown in Figure 4A, in 2030, SO_2_, NO_X_, PM_2.5_, and VOC (volatile organic compounds) emissions will decrease by 22.3%, 19.2%, 14.7%, and 6.9% on the whole in the 15D_Base scenario comparing to BAU_Base. Furthermore, the reduction effect of realizing the 1.5°C goal will be more pronounced in 2050, correspondingly amounting to 34.6%, 46.6%, 19.4%, and 13.6% on the whole. It is worth noting that, except for PM_2.5_, the effect of implementing end-of-pipe technologies in decreasing SO_2_, NO_X_, and VOC emissions in 2050 will be lower than that in 2030, as depict in Figure 4B. These results indicate that, in the future, atmospheric pollutant emission reduction potential from end-of-pipe management will shrink, and pollution alleviation tends to depend more on source treatment, such as optimizing energy consumption structures (Lin et al., 2023). Moreover, driven by the dual targets of carbon reduction and clean air, SO_2_, NO_X_, PM_2.5_, and VOC emissions in 15D_Clean scenario will decrease by 56.2%, 64.8%, 56.3%, and 13.2%, respectively, in 2050, compared with 2020 (Figure S1). The reduction rate for VOC is the smallest, suggesting that VOC emission control is more challenging than other pollutants.^33^ In terms of the emission sectors, as depict in Figure S1, from 2020 to 2050, pollutant emission reductions are mainly driven by the industrial sector. Besides, the residential sector also plays a crucial role in decreasing SO_2_ and PM_2.5_ emissions, as well as the transportation and residential sectors in decreasing VOCs. Overall, by 2050, the industry sector will contribute 53.5%, 40.1%, and 46.6% to SO_2_, NOx, and PM_2.5_ reductions, respectively; residential sector will contribute 43.6% to PM_2.5_ reductions; transportation, and residential sectors will contribute 39.7% and 48.6% to VOCs reductions, respectively.

There are significant differences in pollutant emissions among the six provinces (Figure S2). In terms of the absolute levels, Shandong presents to be the major emitter of NO_X_, PM_2.5_, and VOC. For example, in 2030, NO_X_ emissions in Shandong under the 15D_Clean scenario will amount to be 1.5, 2.0, and 2.4 times higher than that of Henan, Hebei, and Tianjin, respectively. Conversely, the region with the lowest pollutant emissions is Beijing. In terms of the relative changes in pollutant emissions, the top three provinces with particularly prominent reductions during 2030–2050 under the 15D_Clean scenario are Shandong, Henan, and Hebei, with cumulative decline rates of 29.1%, 26.3%, and 30.8%, respectively. Most of these regions are oil refining or heavy industry bases. For Beijing and Tianjin, in despite of the more developed economy obtained, the emission reduction potential appears to be limited as a result of advanced end-of-pipe measures that have already existed. Shanxi, featuring low per capita GDP and high sulfur content of coal, will experience apparent reductions in SO_2_. Furthermore, NO_X_ reductions in the crude oil and coal bases, such as Shandong, Henan, and Hebei, are significant and estimated to exceed 30% of provincial pollutant reductions. The results offer concrete action references for regional atmospheric pollution abatement under the dual targets of carbon reduction and clean air.

### Air quality and human health improvement

**Figure 5:**
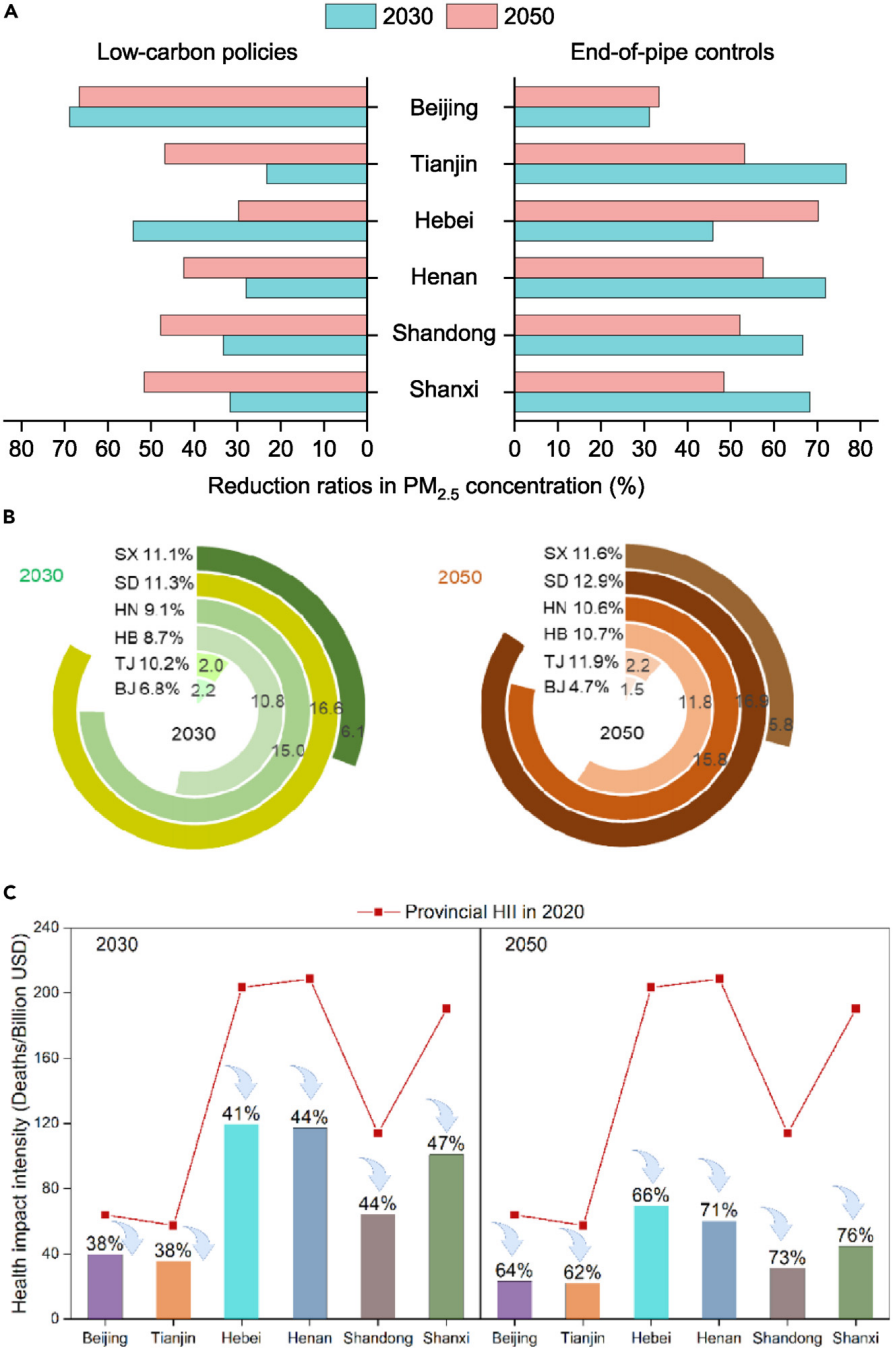
Regional PM_2.5_ concentration reduction effect, PM_2.5_-related deaths changes and health impact intensity in 2030 and 2050 (A) Contribution of low-carbon policies and end-of-pipe controls to reducing PM_2.5_ concentration; (B) avoided deaths [thousand cases] between the BAU_Base and 15D_Clean scenario in 2030 and 2050; (C) health impact intensity [HII] under the 15D_Clean scenario in 2030 and 2050, and the percentages represent the HII reduction rate comparing to 2020).

Implementing low-carbon and end-of-pipe measures is expected to greatly decrease PM_2.5_ concentration.^34^ Under the 15D_Clean scenario, PM_2.5_ concentration of the six provinces will be reduced by 10.6–16.6% and 15.1–23.7% in 2030 and 2050, respectively, compared with their respective levels in 2020 (Figure S3). Furthermore, it is worth noting that evident spatiotemporal disparities exist in the PM_2.5_ concentration reduction effect due to the carbon reduction policies and end-of-pipe technologies, respectively (Figure 5A). Low-carbon policies play key roles in regions such as Beijing, contributing to 68.8% of the total PM_2.5_ concentration reductions in 2030, indicating that synergistic effects generated by carbon reduction to improve air quality surpass the direct benefits obtained from applying end-of-pipe measures. The results are consistent with Wang et al.^35^ On the contrary, in Shanxi, Henan, and Shandong, which have a lot of coal mining and energy-intensive manufacturing, the contribution of synergies in reducing PM_2.5_ concentration is relatively limited (e.g., 28.0–33.3% in 2030). Although the synergistic effects generated by carbon reduction in improving air quality will increase among energy-intensive areas during 2030–2050, end-of-pipe technology is likely to remain dominant in reducing PM_2.5_ concentration, which contributes to 48.4–57.5% of the PM_2.5_ concentration reduction in 2050. Meanwhile, our results present that decarbonizing the energy system is bound to become increasingly important for air quality improvement in the long term, as applying end-of-pipe technologies alone are unable to reach the air quality objectives recommended by WHO in China.

Exposure to PM_2.5_ pollution leads to premature death.^36^ It can be ∖n that regions with high levels of PM_2.5_ concentration and large population density suffer more mortalities (Figure S4). For example, Henan and Shandong will contribute 30.7% and 26.6% to the total number of premature deaths among the six provinces in 2030 under the 15D_Clean scenario. The PM_2.5_-attributable mortalities will be significantly reduced in each region during 2020–2050 with the improvement of air quality. Specifically, the most effectively controlled region is Shandong, where 16.9 thousand (12.9%) mortalities will be avoided in 2050 in the 15D_Clean scenario compared with the BAU_Base scenario (Figure 5B). It is followed by Henan (decreased by 15.8 thousand, 10.6%), Hebei (decreased by 11.8 thousand, 10.7%), and Shanxi (decreased by 5.8 thousand, 11.6%). Beijing produces the smallest decrease among the six regions, with the number of mortalities reduced by only 1.5 thousand (4.7%). Furthermore, the health impact intensity (HII, PM_2.5_-related deaths per unit GDP) is established to examine the economic environmental impact.^37^ As depict in Figure 5C, in 2030, Hebei will have the largest HII (119.6 deaths/billion USD) under the 15D_Clean scenario, followed by Henan (117.3 deaths/billion USD), Shanxi (101.0 deaths/billion USD) and Shandong (64.3 deaths/billion USD). The same order will also appear in other years in the future. It indicates that in these regions, economic output is accompanied by high health costs. In addition, from 2020 to 2050, HII of each region will continuously decrease. Among which, a maximum reduction of 76.4% occur in Shanxi by 2050 under the 15D_Clean scenario compared with 2020 and more than 60% HII reduction occur in other areas. This suggests that the health costs associated with economic output will significantly decline in each region toward meeting the climate and clean air goals.

### Cost-benefit analysis

**Figure 6:**
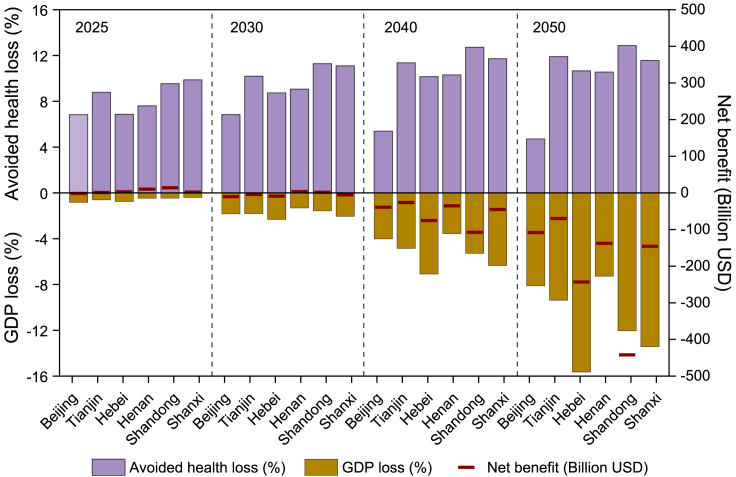
Changes in total cost-benefit for each province under the 15D_CLE scenario compared with the BAU_Base scenario

Figure S5 presents the GDP value under the BAU and 15D scenarios during 2020–2050. Achieving climate goals will affect the provision of social factors, leading to different levels of GDP loss.^38^ In this study, the GDP in BAU will reach 495.1 billion USD (Shanxi)—2065.4 billion USD (Shandong) in 2030. As depict in Figure 6, comparing with BAU, the 15D scenario will lose 1.3% (Henan)—2.3% (Hebei) of GDP in 2030, therefore the CO_2_ reduction cost amounts to 51.2 USD/ton (Beijing)—406.3 USD/ton (Shandong). Furthermore, in 2050, the GDP in BAU will reach 829.6 billion USD (Tianjin)—4161.4 billion USD (Shandong). Realizing the 1.5°C target will lead to a GDP loss ranging from 7.3% (Henan)—15.7% (Hebei), and the carbon reduction cost will amount to 421.2 USD/ton (Hebei)—1498.8 USD/ton (Beijing). Hebei will suffer the most significant economic impact with the implementation of carbon reduction approaches, with a 2.3% and 15.7% loss of GDP in 2030 and 2050, respectively, followed by Shanxi (2.1% and 13.4%) and Shandong (1.6% and 12.1%). The main reason for these high levels of GDP loss may be due to their high CEI of the industrial sector, as strict CO_2_ reduction limit is likely to decrease industry capacity, thus affecting economic outputs. Therefore, to avoid excessive effects of CO_2_ reduction strategies on economy, these provinces need to develop more industries with low resource dependence.

In terms of the end-of-pipe control cost, results show that end-of-pipe costs under the 15D scenario will be lower than the corresponding BAU scenario (Figure S6). This is mainly due to that the carbon reduction pathways will reduce the initial pollutant emissions through optimizing energy and industrial structures, thus saving the end-of-pipe costs. This finding is consistent Lin et al.^39^ In this study, compared to BAU_Base, savings of 152.5 million Euro (Beijing)—2254.2 million Euro (Shandong) in pollution control cost will be achieved under the 15D_Base scenario in 2050. The 15D_CLE scenario further strengthens end-of-pipe technology comparing with 15D_Base, requiring additional costs ranging from 1132.1 million Euro (Beijing) to 14054.2 million Euro (Shandong) in 2050. Besides, health-related economic benefits obtained through air quality improvement depicts in Figure S7. Due to the GDP loss under climate goals are significantly higher than the pollution control cost under clean air targets, cost-benefit analysis in this study is conducted by comparing the GDP loss and health-related economic benefits. As shown in Figure 6, in 2025, the 15D_CLE circumstance brings positive monetary benefits comparing with BAU_Base, amounting to 1.21 billion USD (Tianjin)—14.1 billion USD (Shandong). Shandong experiences the largest economic benefit in 2025, followed by Henan (10.3 billion USD) and Hebei (3.0 billion USD). It is worth noting that Beijing experiences a negative benefit in 2025, which is mainly due to that its pollution abatement potential is limited. After 2030, carbon reduction cost presents exponential upward trends, and appears to be larger than the benefit in each region. Comparing to BAU_Base, net economic benefits under the 15D_CLE scenario present to be negative, ranging from −70.0 billion USD (Tianjin) to −442.2 billion USD (Shandong) in 2050 among the six provinces.

## Discussion

Achieving climate goals requires profound transformations in energy system.^40^ At the national level, Zhang et al. (2021) put forward that by 2060, non-fossil fuels under the carbon neutrality goal will account for 86% of China’s primary energy consumption, of which solar and wind occupy the largest shares. From the perspective of sectors, Ma et al. (2023) evaluate that in 2060, energy consumption in China’s rural residential sector will decline from 79 million tons of standard oil (dominated by coal and traditional biomass) in the baseline scenario to 26 million tons (dominated by electricity) in the carbon-neutral scenario. At the regional level, Liu et al.^32^ point out that the electricity’s share in total energy consumption in the Great Bay Areas (GBA) of China will rise from 36.6% under BAU to 79.3% under the 15D scenario in 2050. For comparison, our evaluation demonstrates that among the six regions, proportion of electricity in final energy under the 15D circumstance will reach 20.8% (Hebei)—53.7% (Beijing) in 2050. It is reasonable that the electrification rates under the 15D scenario in this study are lower than the results for GBA, as GBA is the front-runner in harmonious development of economy and environment.^41^^,^^42^ Moreover, decreasing final energy consumption and bringing forward the corresponding peaking years are needed in order to realize climate goals, which undoubtedly decreases carbon emission.^43^^,^^44^ Our evaluation shows that significant CO_2_ reductions are observed in the 15D circumstance with the peak value occurring between 2020 and 2025 and decreasing by 17.8–26.4% among the six provinces compared with BAU. It accords with other studies. For instance, Ma et al.^45^ indicates that energy-related CO_2_ emissions in China will peak in 2025 with (7608.7, 7634.5) million ton, which will be 5 years before the 2030 goal and likely to decrease the costs toward carbon neutrality.

The low-carbon transformation in energy system also has great significance to alleviate air pollution, apart from mitigating climate change.^46^^,^^47^ For example, it has been evaluated that the carbon neutrality pathway is beneficial to reduce SO_2_ and NO_X_ emissions by 36% and 43%, respectively, in 2050, in Shaanxi.^39^ It is comparable to our results, where SO_2_ and NOx emissions in 2050 under the 15D circumstance among the six provinces are expected to decrease by 24.0–44.0% and 34.4–57.2%, respectively, compared with BAU. Furthermore, SO_2_ and NO_X_ present higher levels of synergies along with CO_2_ reductions than that of PM_2.5_, as has been concluded in Liu et al.^32^ The reductions in pollutant emissions under climate scenarios will undoubtedly improve air quality.^48^ Zhang et al.^49^ find that, in low-carbon scenarios, PM_2.5_ concentration in China will decrease to below 35 μg/m^3^ in 2030. Besides, our results show that the potential of end-of-pipe management to decrease PM_2.5_ concentration continue to shrink in the future, while the contribution of low-carbon strategies will gradually amplify. This is in accordance with Shi et al.^33^ Aforementioned findings suggest that the decarbonization pathways are of great importance in the battle against atmospheric pollution and the overall synergies ought to be considered when issuing green low-carbon development strategies.^50^ Furthermore, air quality improvement under climate goals benefits human health.^51^^,^^52^ It has been estimated that 1.6 million mortalities related to PM_2.5_ exposure will be avoided in China by 2050 under the 15D scenario.^53^ Consistent with Xie et al.^54^ which demonstrates that carbon reduction poses great positive effects on air quality and health improvement in Eastern China, this study shows that regions featuring high population density and heavy industry benefit more in terms of avoiding PM_2.5_-related mortalities with the implementation of green low-carbon strategies.

Excess emissions of CO_2_ and other GHGs are the leading cause of climate change, so carbon emissions also have negative externalities. The carbon emission reduction policy decomposes the emission reduction target into various sectors and the imposition of energy constraints on sectors affects the supply of the whole social factors, resulting in varying degrees of changes in output, investment, and consumption, thus impacting the whole economic system. The six provinces in this study will suffer a GDP loss of 7.3–15.7% in 2050 under the 15D scenario. It accords with previous assessments. For example, Duan et al.^55^ points out that the economic loss of realizing the 1.5°C target in China ranges from 2.3% to 10.9% in 2050. Lin et al.^39^ conclude that the GDP loss accompanied with carbon neutrality in Shaanxi is as high as 18.9%. Moreover, it has been documented that the carbon reduction costs far exceed the air pollution control costs.^54^ Our estimation suggests that the health-related economic benefits do not offset the carbon reduction costs toward the 1.5°C goal after 2030. Many studies have assessed the benefit-costs under certain climate scenarios.^56^ For example, Xie et al.^57^ evaluate the net benefits for the Asian countries to achieve the 2°C target through comparing carbon reduction costs with health-related economic benefits due to air quality improvement. It finds that the carbon reduction costs will be the highest in China, with a benefit/cost ratio amounting to 1.5 in 2050 (the ration for Asia reaches 3.0). Markandya et al.^58^ demonstrate that the additional costs toward a 1.5°C goal from the 1.5°C goal tend to be higher than the incremental in health co-benefit worldwide (except for India). Xie et al.^54^ find that under the 2°C scenario, the carbon reduction costs and health synergies among different regions in China vary significantly. Zhang et al. (2021) conclude that the health co-benefits associated with PM_2.5_ exposure will be higher than mitigation costs under China’s carbon neutrality goal. Lin et al. (2023) point out that the rapidly rising cost of carbon emission reduction under the carbon neutral goal after 2035 will far outweigh the environmental health benefits in Shaanxi and the net loss of total socioeconomic benefits was 4.5 billion USD in 2035. Moreover, it is worth noting that due to the high level of carbon reduction costs, it would be appropriate that policymakers develop more market-based policies and instruments (such as carbon emission trading policy), to substantially reduce the costs of capital toward low-carbon transition.

### Conclusions

The paper combines the IMED|CGE, GAINS, and IMED|HEL model to comprehensively evaluate how the carbon neutral and clean air policies affect the Beijing-Tianjin-Hebei-Henan-Shandong-Shanxi area. Systematical predictions for energy consumption and structures, carbon and atmospheric pollutant emission paths, air quality and related health effects, and cost-benefits are performed to clarify regionally disproportional outcomes, providing decision supports for adjusting and formulating future green low-carbon strategies.

Achieving the 1.5°C target requires accelerating decreasing energy consumption, especially for fossil fuels. In the 15D scenario, all the six provinces will experience energy consumption with the peaking value 22.6–38.9% lower, an energy peaking time occurring during 2020–2025, and a proportion of electricity consumption significantly increased. The energy-related carbon reduction effect is most significant in Shandong, amounting to be decreased by 74.6% in 2050 toward the 1.5°C goal, followed by Hebei (74.5%) and Shanxi (71.0%) accordingly. At sectoral level, province with the largest CO_2_ reductions in power generation is Shandong, and Hebei experiences great carbon reductions in metal smelting sector. Our results also indicate that in the future, the pollutant emission reduction potential of end-of-pipe measures is continuously shrinking, and will turn to depend more on the carbon reduction pathways. Moreover, at regional level, provinces of Shandong, Henan, and Hebei, most of which are oil refining or heavy industry bases, will experience particularly prominent pollutant reductions driven by dual targets. Overall, the government needs to continue strengthening the landing of actions such as energy structure transformation in the future, and give priority to departments and areas with high energy consumption and difficulty in carbon reduction promotion according to local conditions.

Transitioning toward the carbon-neutral energy system decreases PM_2.5_ concentration, especially in municipalities such as Beijing. And the synergistic effect will gradually amplify as time goes by in Shanxi, Henan, and Shandong, which have massive coal mining and energy-intensive manufacturing. Besides, it is found that HII of each province will decrease significantly from 2020 to 2050. Among which, a maximum reduction of 76.7% occur in Shanxi by 2050 under the 1.5°C goal and more than 60% HII reduction occur in other regions, suggesting that the health costs associated with economic output gradually decline as climate goals met. In addition, Hebei experiences the largest GDP loss by 15.7% GDP in 2050 toward realizing the 1.5°C target, followed by Shanxi (13.4%) and Shandong (12.1%). When comparing the health-related economic benefits with policy costs, the 15D_CLE scenario obtains positive benefits before 2030 when comparing with BAU_Base. However, after 2030, the carbon reduction costs show an exponential upward trend, and presents to be larger than the health-related benefit in each province. Therefore, new revenue growth points should be urgently explored and more zero-carbon and negative-carbon technologies should be developed through green technology innovation when implementing carbon reduction policies to decouple economic development from energy consumption and carbon emissions.

Our result highlights the importance of understanding heterogeneous consequences in energy, environment, health, and economy under climate change mitigation and clean air goals, which can provide decision-making references for promoting coordinated implementation of pollution alleviation and carbon reduction based on local conditions. Under the goal of carbon neutrality, the energy system needs to be deeply transformed to a clean one characterized by low-carbon and zero-carbon. The transition can reduce air pollutant emissions and bring considerable health benefits, which also contributes to achieving a sustainable economy along with it. When selecting appropriate measures, we should pay attention to the degree of synergies between reducing pollutant and carbon emissions while strengthening health-oriented synergies in addressing air pollution and climate change so as to promote meeting the carbon neutrality, clean air, and health goals simultaneously.

### Limitations of the study

Some limitations must be acknowledged. Firstly, we do not account for other different health endpoints associated with air pollution, such as dementia cases. Therefore, our estimated health benefits induced by air pollution alleviation under climate goals may be understated. Secondly, we estimate the health outcomes related to clean air co-benefits while ignoring the climate-related heat mortality, heat-related labor productivity loss, and climate-related extreme weather events of climate action (Zhang et al., 2023). It may lead to underestimating the benefits of climate actions. Thirdly, the monetary health benefits and carbon reduction costs under climate goals are predicated for the Beijing-Tianjin-Hebei-Henan-Shandong-Shanxi region in this study. However, given that different provinces in China feature different characteristics in terms of energy consumption structure, population distribution, and so on, the overall benefits obtained through achieving climate goals may differ and therefore should be evaluated accordingly.

## STAR★Methods

### Key resources table

**Table:** 

REAGENT or RESOURCE	SOURCE	IDENTIFIER
**Deposited data**		

Provincial-level Energy Balance Table	China Energy Statistical Yearbook	http://www.stats.gov.cn/tjsj/tjcbw/
Input-output table	National Bureau of Statistics of China (NBS)	Input-output Tables of China

**Software and algorithms**		

IMED|CGE	IMED|CGE	http://scholar.pku.edu.cn/hanchengdai/imedcge
GAINS	GAINS	https://gains.iiasa.ac.at/gains4/ASN/
IMED|HEL	IMED|HEL	http://scholar.pku.edu.cn/hanchengdai/imedhel

### Resource availability

#### Lead contact

Further information and requests for resources and reagents should be directed to and will be fulfilled by the lead contact, Yang Xie (xieyangdaisy@buaa.edu.cn).

#### Materials availability

The study did not generate new materials.

#### Data and code availability

• This paper analyzes existing, publicly available. These accession numbers for the datasets are listed in the key resources table.
• All custom codes have been deposited on the related website. Links are listed in the key resources table.
• Any additional information required to reanalyze the data reported in this paper is available from the lead contact upon request.

### Method details

#### Modelling framework

This research links the IMED|CGE, GAINS and IMED|HEL model to examine the interactions of energy, environment, health and economy towards achieving the carbon neutrality and clean air goals in Beijing-Tianjin-Hebei-Henan-Shandong-Shanxi region of China. Overall framework depicts in Figure S8. Firstly, influences of achieving climate target on energy consumption, associated carbon emissions and economy are estimated through applying IMED|CGE. Secondly, energy consumption of different sectors and types simulated is fed into GAINS, as well as end-of-pipe technology and corresponding penetration share, so as to estimate emissions of SO_2_, NO_X_, PM_2.5_ and VOCs, PM_2.5_ concentration, and pollution abatement cost. Then, input the PM_2.5_ concentration into IMED|HEL to calculate mortalities and related health loss. Finally, the carbon reduction cost, pollution control cost and health benefits are incorporated to assess the total socioeconomic benefits.

The IMED|CGE model has been extensively applied to simulate the economy evolution and the dynamic interactions among different industries under pre-defined scenarios.^32^^,^^59^ It includes detailed descriptions of energy-intensive and high-emission industries (Table S5), and can be used to calculate how energy consumption, CO_2_ emissions and GDP changes towards realizing different climate targets.

The GAINS model includes several modules: emission scenarios, activity paths (industry, residential combustion, transportation, etc.), end-of-pipe technologies and control costs. It has been applied to assess atmospheric emissions, PM_2.5_ concentration and monetary costs with the implementation of different clean air actions.^60^^,^^61^ All analyses conducted for this study are based on the meteorology and boundary conditions of 2015.

The IMED|HEL model is used to simulate premature deaths and morbidities caused by air pollution based on the exposure-effect function in environmental epidemiology and the monetization evaluation method in environmental economics.^62^ Meanwhile, economic loss associated with mortalities and medical expenditures related to morbidities can be calculated.^63^ This research considers mortalities and associated economic loss resulted from PM_2.5_ pollution.

#### Scenarios and parameter settings

Four scenarios (BAU_Base, BAU_Clean, 15D_Base and 15D_Clean) are set up in this study to analyze the effects of green low-carbon transition in two dimensions (Table S6). The climate perspective consists of a business-as-usual (BAU) scenario and a 1.5°C (15D) one. Specifically, according to the global CO_2_ emission paths under a 1.5°C limit,^64^ provincial CO_2_ emission constraints will be downscaled based on the principle of convergence of per capita emissions. Similar methods have been applied in Liu et al.^32^ The atmospheric pollutant emission aspect includes a frozen (Base) scenario and a current legislation (Clean) one. Specifically, the Base scenario freezes the end-of-pipe removal rates and the Clean scenario assumes the control measures are implemented in line with the government’s strategies.
