# Supplementary material for: Uncovering the differentiated impacts of carbon neutrality and clean air policies in multi-provinces of China
Source: iScience. 2024 May 11;27(6):109966. doi: 10.1016/j.isci.2024.109966 (PMC11144726; doi:10.1016/j.isci.2024.109966)
Supplement: Document S1. Figures S1–S8 and Tables S1–S6 [file mmc1.pdf]

iScience, Volume *27*

Supplemental information

Uncovering the differentiated impacts of carbon neutrality and clean air policies in multi-provinces of China

Meng Xu, Minghao Wang, Mengdan Zhao, Zhixiong Weng, Fan Tong, Yujie Pan, Xin Liu, and Yang Xie

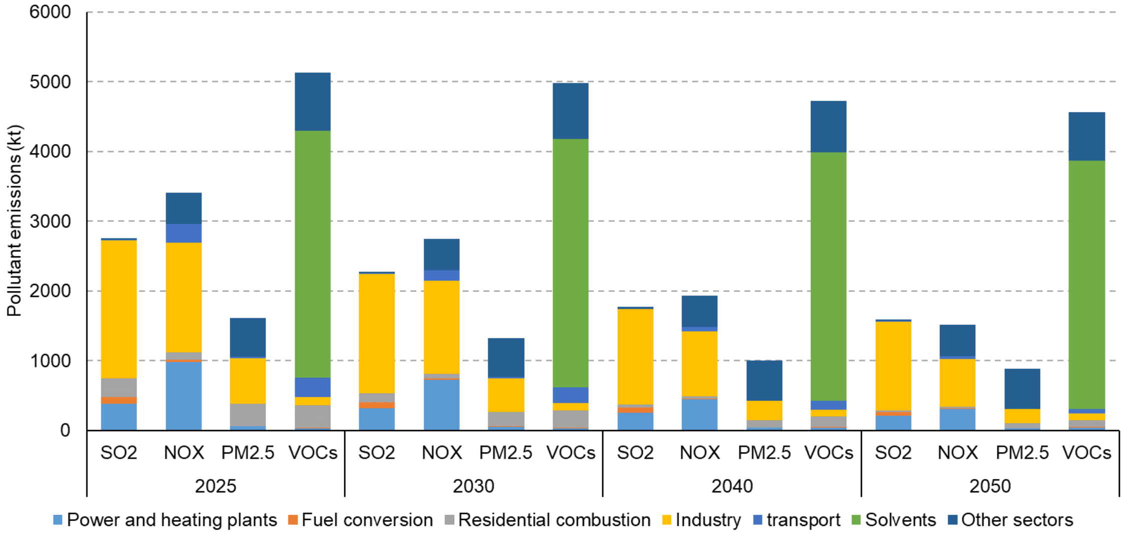

Figure S1. Overall pollutant emissions by sectors in the six provinces under 15D_Clean scenario during 2025-2050, related to Figure 4

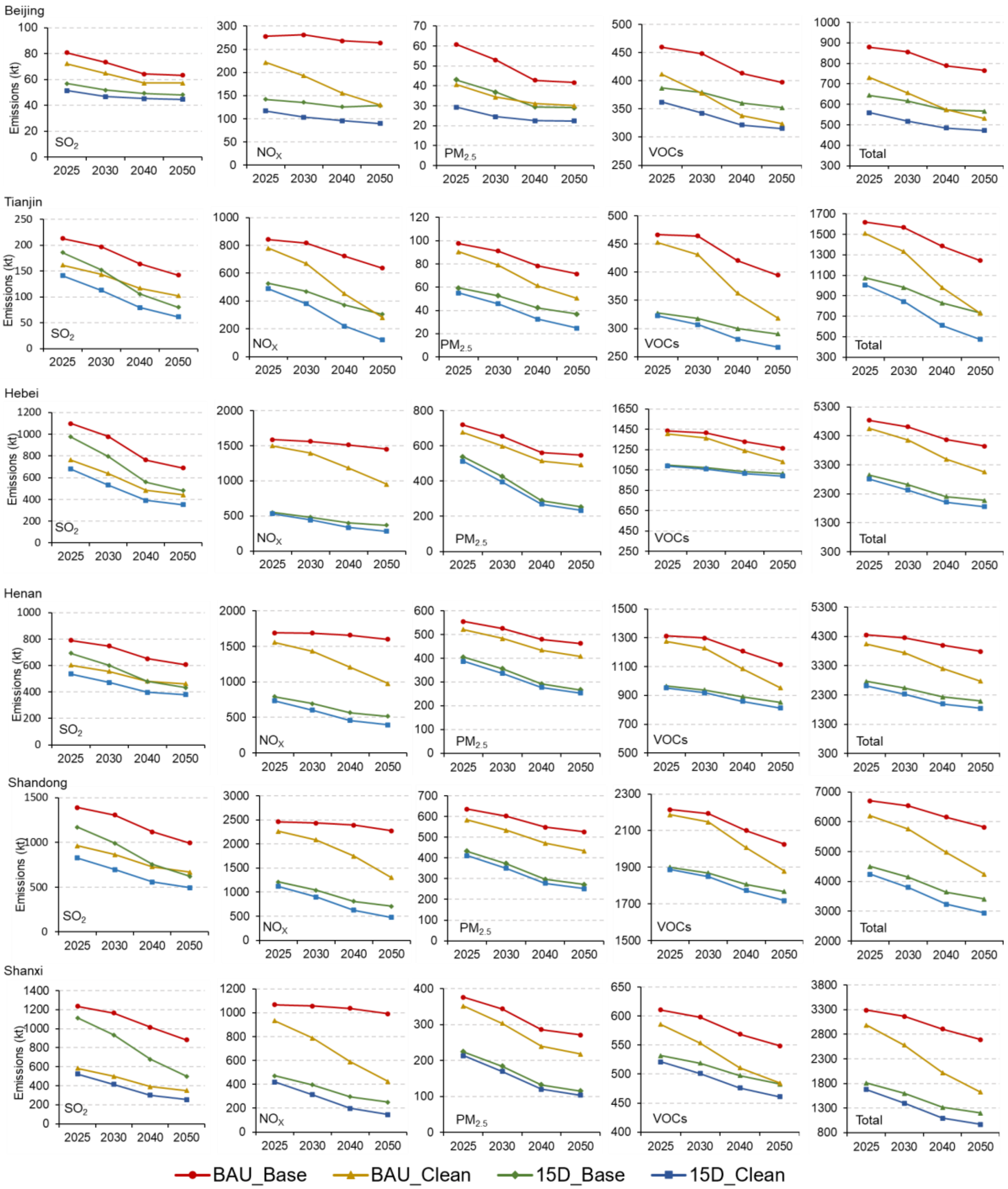

Figure S2. Pollutant emissions of each province under different scenarios during 2025-2050, related to Figure 4

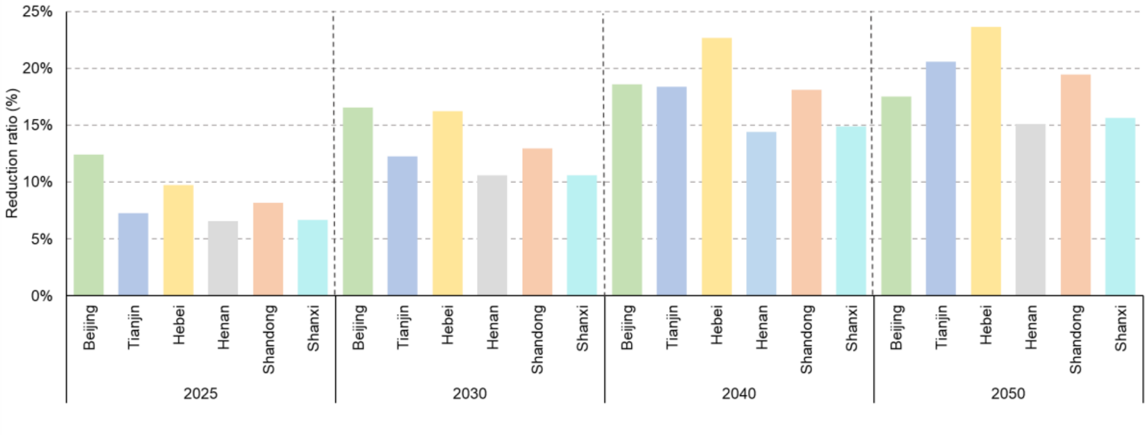

Figure S3. Reduction ratios of $PM_{2.5}$ concentration under the 15D_Clean scenario during 2025-2050 compared with 2020, related to Figure 5

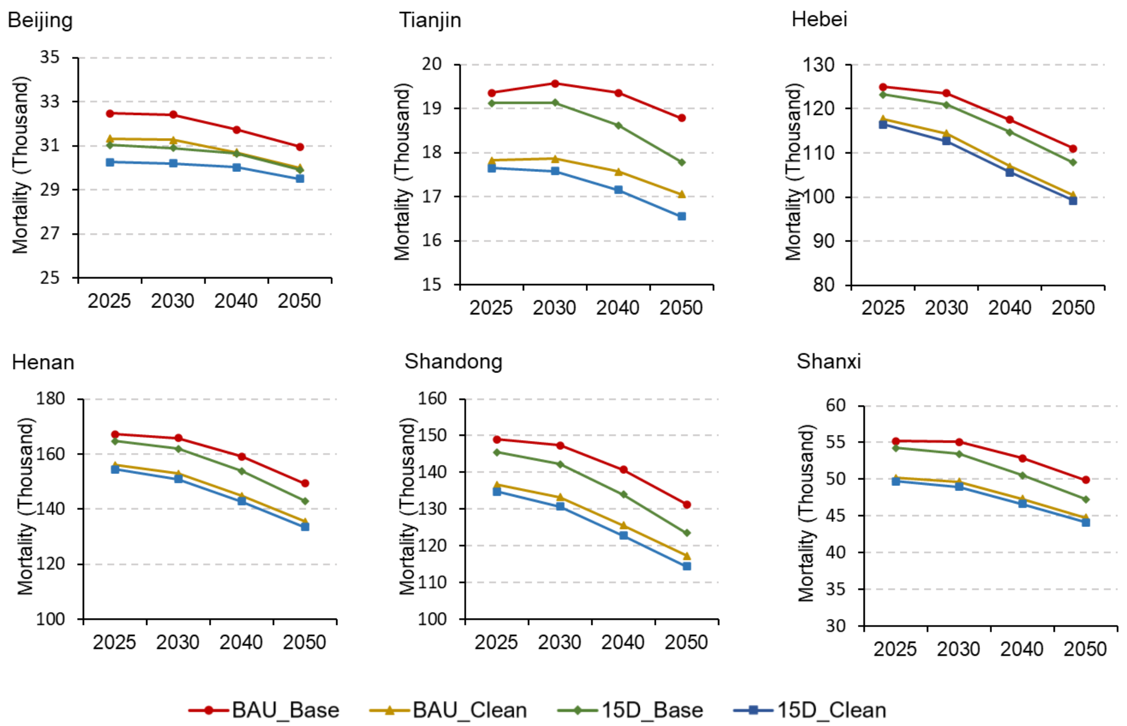

Figure S4. Mortalities due to $PM_{2.5}$ pollution for each province under different scenarios during 2025-2050, related to Figure 5

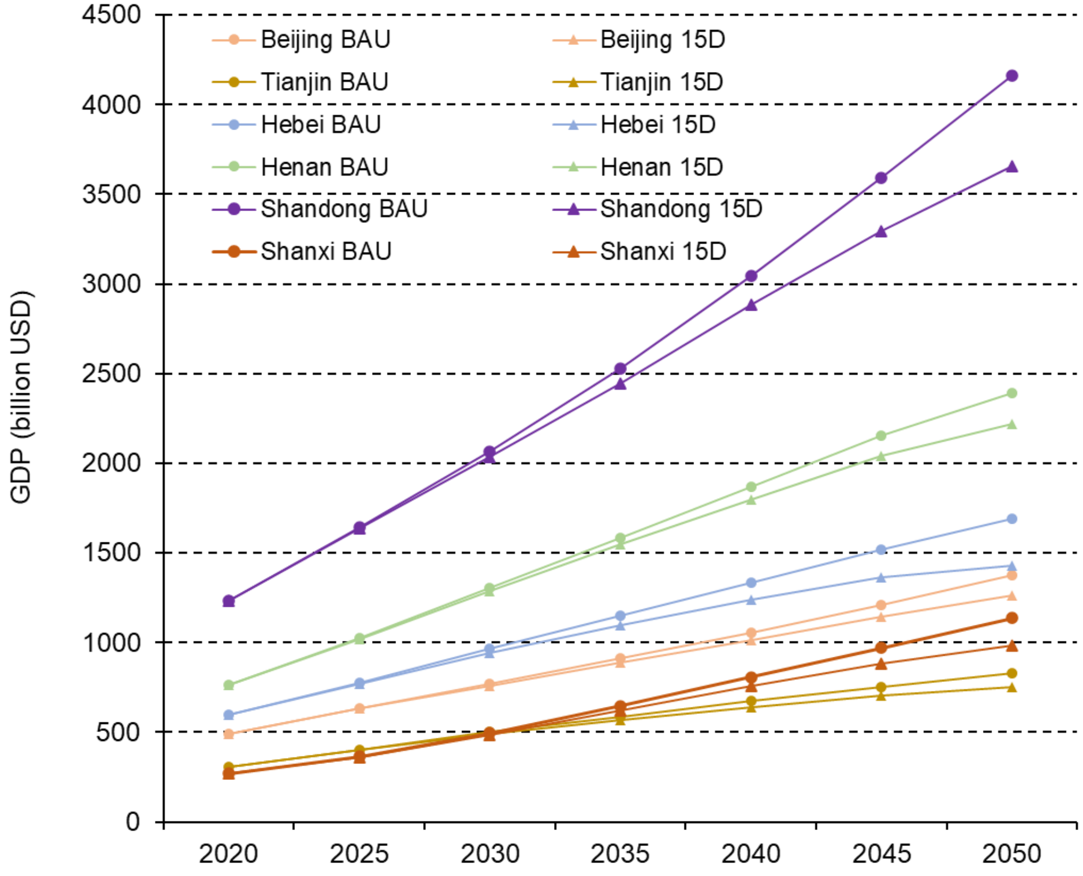

Figure S5. GDP for each province under different scenarios, related to Figure 6

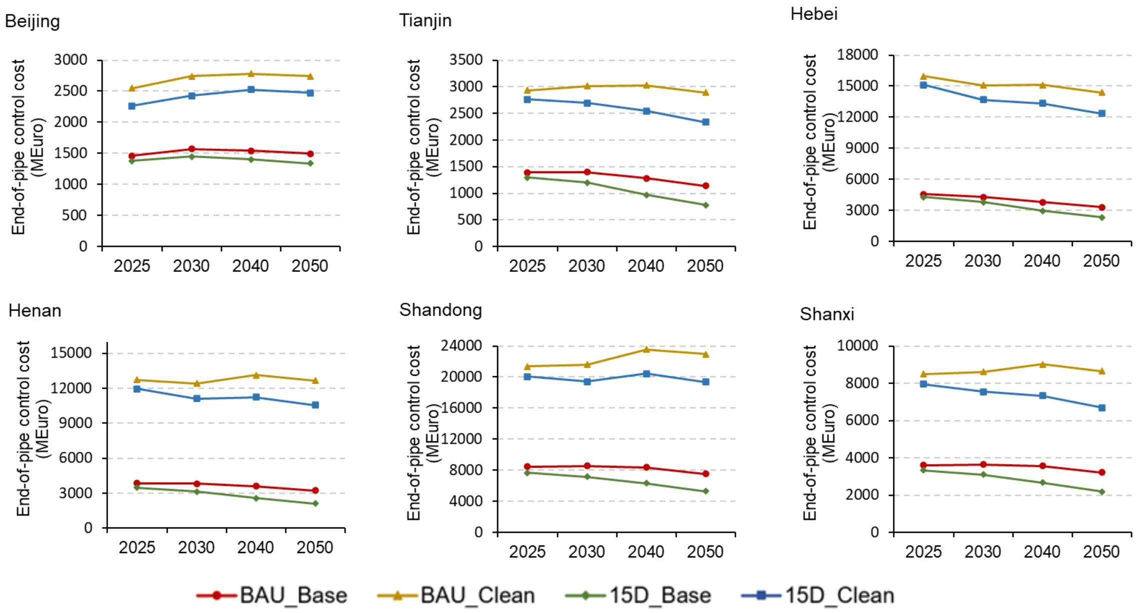

Figure S6. End-of-pipe control costs for each province under different scenarios during 2025-2050, related to Figure 6

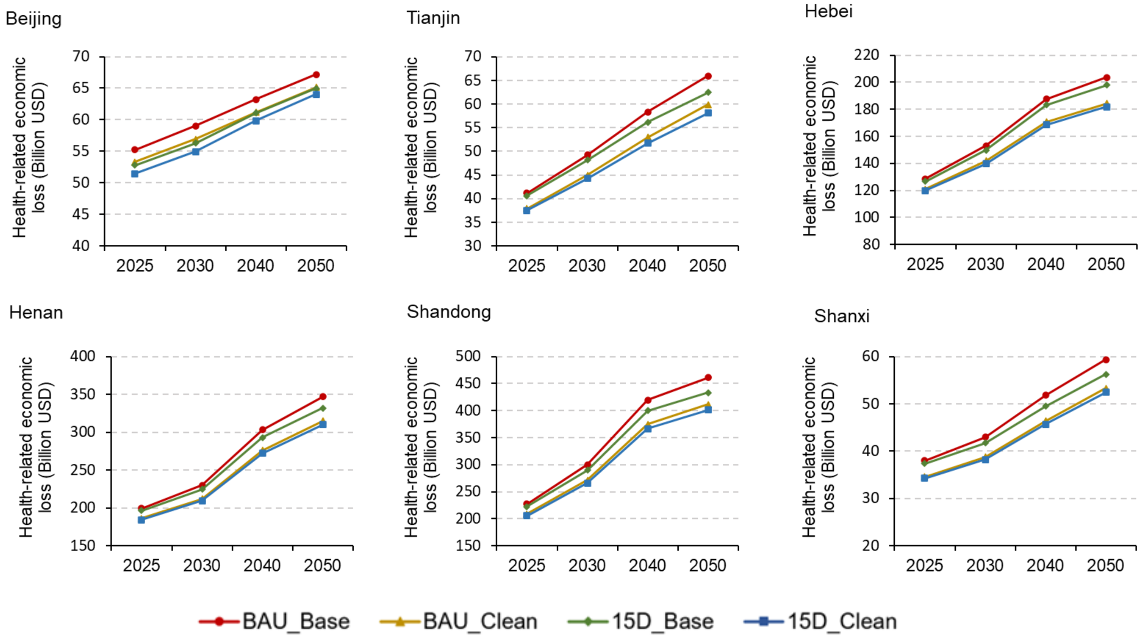

Figure S7. Health-related economic loss due to premature deaths under different scenarios during 2025-2050, related to Figure 6

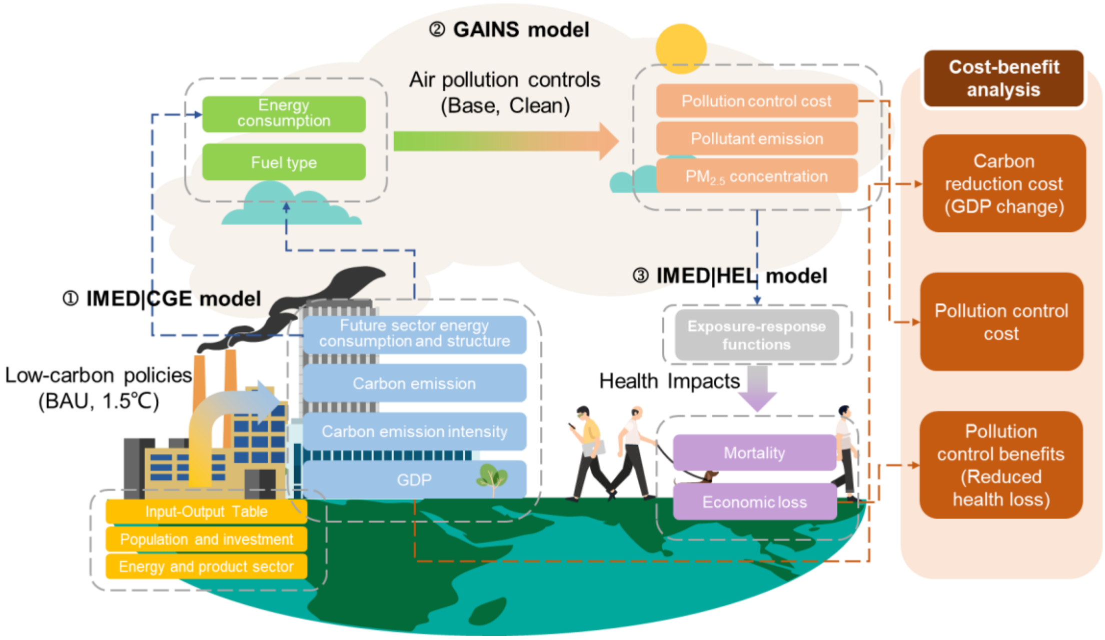

Figure S8. Research framework, related to STAR Methods

Table S1. Provincial energy consumption under different climate scenarios (Mtoe), related to Figure 2

		BAU				15D			
		2025	2030	2040	2050	2025	2030	2040	2050
Beijing	Electricity	14	18	25	33	13	16	19	18
	Coal	3	3	3	2	3	3	2	1
	Gas	27	30	28	23	23	23	18	12
	Oil	21	23	23	20	14	11	5	2
	Coke	0	0	0	0	0	0	0	0
Tianjin	Electricity	10	11	13	14	9	9	8	6
	Coal	23	24	21	16	19	16	10	5
	Gas	18	20	21	18	16	14	10	5
	Oil	19	21	21	17	17	17	12	6
	Coke	7	7	6	4	5	3	2	1
Hebei	Electricity	39	43	47	48	35	35	30	20
	Coal	158	165	154	126	133	115	71	34
	Gas	37	46	60	72	27	24	16	9
	Oil	23	28	34	36	22	25	23	15
	Coke	71	77	78	69	56	49	33	18
Henan	Electricity	40	48	59	65	36	38	36	28
	Coal	133	143	139	116	106	91	55	27
	Gas	22	26	29	29	20	22	21	16
	Oil	30	35	39	37	28	29	25	16
	Coke	22	25	27	26	17	14	10	6
Shandong	Electricity	85	111	167	219	72	80	85	69
	Coal	242	260	250	219	189	161	102	52
	Gas	53	62	71	76	34	32	23	13
	Oil	45	50	51	47	44	47	37	21
	Coke	29	31	32	32	23	19	11	6
Shanxi	Electricity	23	29	42	53	21	23	24	20
	Coal	204	221	208	166	176	159	106	54
	Gas	22	27	30	28	16	13	7	3
	Oil	12	14	13	11	9	7	4	2
	Coke	19	20	18	13	17	17	11	5

Table S2. Provincial carbon emissions during 2020-2050 under different climate scenarios (Mt), related to Figure 3

		BAU				15D			
		2025	2030	2040	2050	2025	2030	2040	2050
Beijing	Power generation	52	56	49	39	52	57	50	33
	Energy supply	18	20	22	21	10	6	1	0
	Textile	0	0	0	0	0	0	0	0
	Chemicals	1	1	0	0	0	0	0	0
	Nonmetal	1	0	0	0	0	0	0	0
	Metal smelting	0	0	0	0	0	0	0	0
	Manufacture	1	1	1	1	1	0	0	0
	Transport	31	34	33	26	22	18	9	3
	Service	10	10	9	9	7	5	3	1
	Household	18	20	19	15	10	6	2	0
	Coal mining	0	0	0	0	0	0	0	0
	Other sectors	2	2	2	2	1	1	1	0
Tianjin	Power generation	72	78	74	62	66	61	41	22
	Energy supply	15	17	17	14	14	13	9	4
	Textile	0	0	0	0	0	0	0	0
	Chemicals	14	15	15	12	11	11	7	3
	Nonmetal	2	2	1	1	2	1	1	0
	Metal smelting	41	41	36	26	30	22	13	6
	Manufacture	9	10	10	8	7	6	4	2
	Transport	13	14	13	9	12	12	8	4
	Service	9	10	12	13	8	8	7	5
	Household	10	13	13	9	8	7	3	1
	Coal mining	0	0	0	0	0	0	0	0
	Other sectors	9	9	9	6	7	7	5	2
Hebei	Power generation	230	230	195	145	210	187	113	50
	Energy supply	44	49	51	47	37	35	26	15
	Textile	1	2	2	2	1	1	1	1
	Chemicals	20	24	28	31	16	15	12	8
	Nonmetal	11	12	13	12	9	8	6	4
	Metal smelting	328	362	386	366	260	228	157	86
	Manufacture	11	13	17	19	9	9	7	5
	Transport	26	29	32	31	25	26	24	16
	Service	18	22	27	30	15	15	13	9
	Household	53	65	78	80	40	37	22	8
	Coal mining	58	62	62	54	39	27	11	3
	Other sectors	16	20	24	27	15	16	16	11
Henan	Power generation	287	305	285	227	241	211	130	65

		BAU				15D			
		2025	2030	2040	2050	2025	2030	2040	2050
	Energy supply	35	39	40	34	27	23	14	7
	Textile	1	1	1	2	1	1	1	1
	Chemicals	17	19	21	22	13	13	11	9
	Nonmetal	13	13	14	13	10	10	8	6
	Metal smelting	97	109	119	113	77	71	60	45
	Manufacture	10	12	15	16	9	9	8	6
	Transport	33	37	40	35	31	32	28	17
	Service	11	14	17	18	10	10	9	7
	Household	24	29	33	30	19	18	11	4
	Coal mining	38	43	47	44	23	17	10	5
	Other sectors	17	20	24	23	16	17	16	10
Shandong	Power generation	574	626	598	513	474	426	286	150
	Energy supply	97	103	99	87	82	74	48	24
	Textile	5	6	6	7	3	2	2	1
	Chemicals	28	30	30	31	17	14	10	6
	Nonmetal	19	19	20	19	11	8	5	2
	Metal smelting	138	150	160	160	93	77	49	26
	Manufacture	24	26	29	32	15	12	9	6
	Transport	50	54	49	39	48	49	37	19
	Service	18	21	23	25	14	14	12	8
	Household	42	49	51	50	36	35	22	8
	Coal mining	40	46	52	53	21	15	8	4
	Other sectors	18	19	18	16	16	16	13	7
Shanxi	Power generation	255	270	255	207	229	208	144	76
	Energy supply	101	114	105	81	76	63	38	16
	Textile	0	0	0	0	0	0	0	0
	Chemicals	12	17	22	24	10	10	8	4
	Nonmetal	8	10	11	9	7	6	4	2
	Metal smelting	94	102	89	63	82	75	48	22
	Manufacture	5	7	8	7	5	4	3	2
	Transport	24	27	23	17	19	16	9	4
	Service	14	17	19	19	11	11	8	5
	Household	17	19	18	13	10	7	2	1
	Coal mining	23	27	31	31	17	14	8	4
	Other sectors	10	13	16	16	8	8	7	4

Table S3. Pollutant reduction effect between the BAU_Base and 15D_Base scenario (%), related to Figure 4

		Beijing	Tianjin	Hebei	Henan	Shandong	Shanxi
2030	SO_2	29	23	19	19	24	20
	NO_X	31	18	11	15	14	25
	$PM_{2.5}$	35	13	9	8	11	12
	VOC	16	7	4	5	2	7
2050	SO_2	24	44	30	29	38	43
	NO_X	51	56	34	39	43	57
	$PM_{2.5}$	28	30	10	12	18	20
	VOC	18	19	10	15	7	12

Table S4. Pollutant reduction effect between the 15D_Base and 15D_Clean scenario (%), related to Figure 4

		Beijing	Tianjin	Hebei	Henan	Shandong	Shanxi
2030	SO_2	10	26	33	21	30	56
	NO_X	46	43	68	58	57	60
	$PM_{2.5}$	29	42	34	30	34	44
	VOC	9	29	22	25	14	9
2050	SO_2	7	23	27	12	20	49
	NO_X	31	57	70	59	63	66
	$PM_{2.5}$	25	51	52	38	42	53
	VOC	3	16	13	15	9	5

Table S5. Sectoral classification in IMED|CGE, related to STAR Methods

Number	Abbr.	Sector	Number	Abbr.	Sector
1	AGR	Agriculture	16	MPD	Metal products
2	COA	Coal mining and dressing	17	ELP	Electric equipment manufacturing
3	COL	Crude oil	18	OMF	Other manufacturing
4	GAS	Natural gas exploitation	19	WTR	Water production and supply
5	OMN	Other mining	20	ELE	Power generation
6	TEX	Textile	21	GDT	Gas production and supply industry
7	PPP	Papermaking and paper products	22	CNS	Construction
8	FOD	Food production	23	TRL	Rail transport
9	LUM	Wood and furniture manufacturing	24	TRD	Road transport
10	PET	Pereoleum and nuclear fuel processing industry	25	TPL	Transit transport
11	CHM	Chemicals	26	TWT	Water transport
12	ONM	Nonmetal industry	27	TAR	Aviation
13	MCN	Machinery manufacturing	28	TPP	Pipeline transport
14	I_S	Iron steel manufacturing	29	CSS	Services
15	NFM	Non-ferrous metal smelting and processing			

Table S6. Scenario setting with two dimensions of climate mitigation and air pollution control, related to STAR Methods

Scenario	Definition	Climate target	End-of-pipe pollution control
BAU_Base	BAU_Base presumes China will achieve its Nationally Determined Contributions (NDCs) pledges, and the air pollutant end-of-pipe control would remain at the 2020 level.	CO_2 emissions peak in 2030 that in line with NDC pledges	End-of-pipe removal rate control frozen at the 2020 level
BAU_Clean	BAU_Base presumes China will achieve its Nationally Determined Contributions (NDCs) pledges, and deploy strong end-of-pipe control technologies.	CO_2 emissions peak in 2030 that in line with NDC pledges	Current released and upcoming policies
15D_Base	15D_Base is designed to pursue the 1.5 °C climate target and assumes a faster rate of electricity substitution and a higher level of technological progress than BAU. The air pollutant end-of-pipe control would remain at the 2020 level.	1.5 °C	End-of-pipe removal rate control frozen at the 2020 level
15D_Clean	15D_Clean shares the same energy and socioeconomic development with 15D_Base, but will deploy stronger end-of-pipe control technologies.	1.5 °C	Current released and upcoming policies
